# Association between urine and serum estradiol levels in in vitro fertilization cycles

**DOI:** 10.1038/s41598-022-08292-z

**Published:** 2022-03-15

**Authors:** Chokchai Chotboon, Lingling Salang, Pranom Buppasiri, Siriruthai Amnatbuddee, Nuntasiri Eamudomkarn

**Affiliations:** Department of Obstetrics and Gynecology, Faculty of Medicine, Khon Kaen University, Khon Kaen, 40002 Thailand

**Keywords:** Chemical biology, Medical research

## Abstract

To study the correlation between urine and serum estradiol (E2) controlled ovarian hyperstimulation (COH). This is a cross-sectional analytical study that was conducted in a tertiary care hospital. Seventy-seven urine and blood samplings were collected from infertile women who were treated with COH. An electrochemiluminescent immunoassay was performed to evaluate E2 levels between urine and serum samples on the 6th day and the day of ovarian trigger. In addition, the correlations were evaluated between urine E2 level and number of follicles, retrieved, metaphase II oocytes, and fertilization rate. A sub-analysis was performed for age, responding status and BMI. Seventy-seven infertile women were recruited. The medians of serum and urine E2 level levels on the day 6th of ovarian stimulation were 833.20 pg/ml (IQR; 516.90–1371.00) and 3.67 (IQR; 2.84–4.81), respectively. On the day of ovarian trigger, the median of serum E2 level was 2113.00 pg/ml (IQR; 1382.00–3885.00) and urine E2 level (E2/creatinine) was 6.84 (IQR; 5.34–8.70). The correlation between serum and urine E2 level on day 6th was 0.53 and the day of ovarian trigger was 0.59, *p* < 0.001. Moreover, the correlations of urine E2 level on the day of ovarian trigger to number of follicles, number of oocytes retrieved, metaphase II oocytes and fertilization rate were 0.57, 0.58, 0.61, and 0.64 (*p* < 0.001). The urine E2 level was moderately correlated to serum E2, number of follicles growth, oocytes retrieved and fertilization rate.

## Introduction

Assisted reproductive technologies (ART) are widely used for treatment of infertility. To increase the success rate of ART, controlled ovarian hyperstimulation (COH) with gonadotropin administration is used to recruit multiple follicles^1^. However, using convention COH to obtain more follicles increase risk of ovarian hyperstimulation syndrome (OHSS)^2,3^.

Recently, counting ovarian follicular under transvaginal ultrasound and/or serum hormonal assessment, particularly estradiol level is used to predict ovarian response. There are two crucial reasons for monitoring during COH. First, to assist making decision on the appropriate time of trigger final oocyte maturation to increase chance of success rate. Second, to evaluate the risk of over response^4^. Frequent monitoring especially serum hormonal assessment induces psychological stress in patient^5^. Therefore, alternative hormonal measurement such as urine estradiol analysis may be advantage for assessing ovarian response due to easy collection and less invasive.

Based on basic knowledge, serum estrogen was metabolized and secreted via urine into many forms such as urinary estrone metabolites, estriol, 2-hydroxyestrone, 2-methylestrone, 2-methoxyestrone, 2-hydroxyestrone-3-methyl ether, 4-hydroxyestrone, 4-methoxyestrone, 16α-hydroxyestrone and urinary E2 metabolites. Therefore, the urine E2 level is the most relevant to serum E2 level^6,7^. Recently studies have been reported that the serum E2 level is moderately correlated to urine E2 level in pre- and postmenopausal women^8–12^. In COH cycle, there were a few studies that reported the correlation between urine and serum E2. Alper 1994, reported urinary estrone conjugated (E1C) level in women receiving human menopausal gonadotropin (hMG) correlates with serum estradiol (E2) in day of ovarian trigger and the correlation between E1C and E2 was 0.85 (*P* < 0.0001)^8^. However, this study was reported the correlation between serum and urine estrogen only the day of ovarian triggering. From the gap of knowledge, we developed our study to assess the correlation between urine and serum E2 in women treated with COH on day 6th of ovarian stimulation and day of ovarian trigger, and to evaluate the correlation between urine E2 level to the number of follicles, number of retrieved oocytes, number of metaphase II oocytes, and percentage of fertilization.

## Material and methods

### Study participant and study design

A cross-sectional study was conducted. The serum and urine samples were obtained in the infertility clinic between April 2020 and November 2020 from infertile women treated with controlled ovarian hyperstimulation for IVF or ICSI at Srinagarind Hospital, Khon Kaen University. The inclusion criteria were women aged > 20 years-old without contraindication for hormonal treatment. After receiving written informed consents of the participants, urine specimens were collected at the same time of routine blood collections in the morning on the 6th day and the last day of ovarian stimulation. Other information such as the cause of infertility, body mass index (BMI), type of hormone, and follicular sizes were recorded, and the follicular sizes were measured by 2 dimensional (2D) transvaginal ultrasonography. This research was approved by the Khon Kaen University Ethics Committee on Human Research (HE621557).

### Controlled ovarian hyperstimulation protocol

Transvaginal ultrasound was performed for all participants on the second or third day of menstruation in order to identify the confounding factors such as ovarian cyst that may be affect on estradiol level. In the woman with normal transvaginal ultrasound finding, daily gonadotropin injection treatment, the HMG (Menopur, Ferring AG, Germany) or rFSH (Gonal-F, Merk, France) was started on the 2nd or 3rd day of menstruation. The dosage of gonadotropin stimulation using depended on antral follicle count measurement under ESHRE guideline^13^. According to type of gonadotropin using, the protocol depended on physician decision. Long-acting rFSH treatment at a subcutaneous dose (100–150 mcg) of corifollitropin alpha (Elonva, MSD, USA) was injected on the second or third day of menstruation. The treatment continued from day six of stimulation with rFSH or HMG and the dosage of gonadotropin was adjusted following the size of follicle growth under EHSRE guideline^13^. The follicle size was more than 10 mm. the gonadotropin was continue in the same dosage. In contrast, if dominant follicles size was less than 10 mm, we would add 75 unit of gonadotropin. The GnRH antagonist (Orgalutran, MSD, USA) was used daily until triggering occurred when the leading follicles reached a mean diameter of 14 mm. The ovulation was triggered by human chorionic gonadotropin (HCG) or GnRH agonist alone or dual trigger (combined GnRH agonist with HCG) in case of > 2 dominant follicles of > 17 mm.

### Estradiol analysis

Both urine and serum E2 levels were analyzed by an Electrochemiluminescent immunoassay (ECLIA). The ECLIA at the KKU center is Elecsys Estradiol III Cobase 801 that employs a competitive test principle using two monoclonal antibodies specifically directed against 17-β E2 and standardized with ID-GC/MS Calibration monthly.The coefficient of variation was 3.6%. The duration of the automated assay was 18 min, calibrated with an internal quality control daily and external quality control monthly. For control the confounding factor, urine E2 level (pg/ml) was adjusted its level with urine creatinine (mg/dl) as the urine E2-creatinine ratio (E2/creatinine) to correct the glomerular infiltration rate.

Serum and urine collection: Ten mL of blood samples were collected in plain tubes that included coagulation activator followed by followed by centrifugation at 1720*g* for 10 min; serum was collected and stored at 4 °C until the assay was performed within 48 h after sample collection. Fifty mL of urine specimens were collected in the morning on the same day as blood collection. Urine specimens were subjected to centrifugation at 430*g* for 10 min; supernatants were collected and stored at 4 °C until the assay was performed, which was within 48 h after the sample collection. Urine and serum E2 levels were measured by Electrochemiluminescent immunoassay (ECLIA)^15^. Our study was the first study that using ECLIA to evaluate urine E2. Therefore, we developed pilot study for testing accuracy of this method. We applied 10 serum and urine E2 sampling from other women who received COH regimen on day 6th of ovarian stimulation and day of ovarian trigger. The correlation (Rho) between urine and serum E2 level on day 6th of stimulation and day of ovarian trigger was 0.67 and 0.81, respectively.

### Data analysis

Based on the result of Maskarinec et al.^10^, with a 10% dropout rate, 77 participants were needed. The general appearances of participants were analyzed by SPSS V.23 in terms of mean and standard deviation (SD). The correlations between urine E2 to serum E2, ultrasonographic follicles, retrieved oocytes, metaphase II oocytes and fertilization rate were analyzed by Spearman’s correlation. A *p* value < 0.05 indicated statistical significance.

### Sample size

There are not any studies comparing between urine and serum estradiol in women treated with assisted technology. The study of Maskarinec et al.^10^ was cited to calculate the sample size. Maskarinec G’s study was the largest study, included Asian and young population, studied about correlation between urine and serum estradiol of premenopausal women that was 0.35, *p* < 0.001. We provided type I error as 0.05 and 80% of power. A total of 77 patients were recruited in this study included 10% dropouts. This study used this formular of sample size calculation.

### Ethical consideration

The Human Research Ethics Committee of Khon Kaen University reviewed and approved the study per the Helsinki Declaration and the Good Clinical Practice Guidelines (HE621557).

### Consent for publication

All of the authors consent to publishing and hereby grant the Publisher exclusive license of the full copyright.

## Results

A total of 77 infertile women were included. Their mean age was 35.81 ± 4.49 years, BMI was normal (22.63 ± 3.54 kg/m^2^) and most of them were primarily infertile (89.61%). The three main causes of infertility were unexplained 35.6%, male factor 29.8% and tubal factor 23.4% (Table 1). The most common type of gonadotropin injection was daily human menopausal gonadotropin (HMG) 36.36%, followed by recombinant FSH (rFSH) 35.07% and combined long acting rFSH (corrifollitropin alpha) followed by rFHS or HMG 28.57%. The average day of gonadotropins injection was 9 days, and the average of the gonadotropin injection (not include Corrifollitropin alpha dosage) was 2495.91 units. In gonadotropin releasing hormone (GnRH) antagonist types, half of the participants (51.95%) were given the dual trigger (combined GnRH antagonist with HCG) while 36.36% and 11.69% were triggered by GnRH-antagonist alone or HCG alone. The median of retrieved oocytes and metaphase II oocytes were 9 oocytes (IQR; 5–18) and 7 oocytes (IQR; 4–15). The numbers of 2PN were 5 (IQR; 3–10), cleavage 5 (IQR; 2–9, and blastocyst stages 4 (IQR; 1–6).

**Table 1:** Baseline characteristics.

**Baseline cycle characteristics (mean ± SD)**	
Age (years)	35.81 ± 4.49
BMI (kg/m^2^)^a^	22.00 (17.26–37.13)
**Parity (%)**	
Primary infertility	69 (89.61)
Secondary infertility	8 (10.39)
**Cause of infertility (%)**	
Unexplained	27 (35.06)
Male factors	23 (29.87)
Tubal factors	18 (23.38)
Ovulation disorders	6 (7.79)
Uterine factors	3 (3.90)
AFC^a^	7 (2–25)
**Gonadotropin types (%)**	
HMG	28 (36.36)
rFSH	27 (35.07)
Corifollitropin alpha followed by rFSH or HMG	22 (28.57)
Days of gonadotropin injection (days)^a^	10 (9–10)
**Gonadotropin (mean ± SD)**	
Cumulative gonadotropin dose; HMG or rFSH (IU) (not include Corrifollitropin alpha dosage)	2495.91 ± 600.81
Long-acting gonadotropin (Corrifollitropin alpha) (mcg)	127.27 ± 25.48
Days of GnRH antagonist injection (days)^a^	4 (3–5)
**Hormone for trigger ovulation (%)**	
Combined GnRH agonist and HCG	40 (51.95)
GnRH agonist	28 (36.36)
HCG	9 (11.69)
Number of retrieved oocytes^a^	9 (5–18)
Number of metaphase II oocytes^a^	7 (4–15)
**Fertilization rate**^**a**^	
Number of 2PN stage	5 (3–10)
Number of cleavage stage	5 (2–9)
Number of blastocyst stage	4 (1–6)

*rFSH* recombinant follicle stimulating hormone, *HMG* human menopausal gonadotrophin, *HCG* Human Chorionic Gonadotropin, *GnRH* Gonadotropin releasing hormone, *PN* pronuclei.

^a^Data presented as median and interquartile range (IQR).

**Figure 1:**
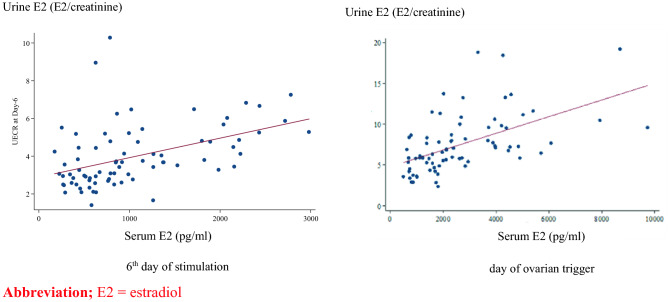
Correlations between urine and serum E2 concentration on 6th day of stimulation and day of ovarian trigger. *E2* estradiol.

The median serum E2 levels were 833.20 pg/ml (IQR; 516.90–1371.00) on the 6th day and 2113.00 pg/ml (IQR; 1382.00–3885.00) and on the last day of ovarian stimulation. While the median urine E2 levels (E2/creatinine) were 3.67 (IQR; 2.84–4.81) on the 6th day and 6.84 IQR; (5.34–8.70) on the last day of ovarian stimulation (Table 2). In addition, the urine E2 level was correlated with serum E2 level at 0.53 (*p* < 0.001) on the 6th day of ovarian stimulation and 0.59 (*p* < 0.001 on the day of ovarian trigger. Furthermore, Fig. 1 demonstrates the linear correlations between urine and serum E2 level on the 6th day of stimulation and on the day of ovarian trigger.

**Table 2:** Data distribution of urine and serum estradiol.

E2 level	Median (IQR)	95% CI
**Serum estradiol (pg/ml)**		
On 6th day of stimulation	833.20 (516.90–1371.00)	892.39–1210.39
On the day of ovarian trigger	2113.00 (1382.00–3885.00)	2232.59–3075.20
**Urine estradioll (estradiol/creatinine ratio)**		
On 6th day of stimulation	3.67 (2.84–4.81)	3.61–4.34
On the day of ovarian trigger	6.84 (5.34–8.70)	6.71–8.29

In the secondary outcomes, on the 6th day of stimulation, the urine E2 was correlated as Spearman's correlation coefficient rate 0.39 (*p* = 0.001), to sonographic follicles (≥ 10 mm), retrieved oocytes 0.42 (*p* < 0.001), metaphase II oocytes 0.44 (*p* < 0.001), 2PN rate 0.47 (*p* < 0.001), cleavage rate, 0.43 (*p* < 0.001) and blastocyst 0.48 (*p* < 0.001). The day of ovarian trigger showed higher correlations than the 6th day of urine E2 to sonographic follicles (≥ 14 mm) 0.57, retrieved oocytes 0.58, metaphase II oocytes 0.61, 2PN rate 0.64, cleavage rate 0.62 and blastocyst rate 0.57 (*p* < 0.001) as Spearman's correlation coefficients (Table 3).

**Table 3:** Spearman’s correlation coefficients for urine estradiol level in the infertility treatment outcomes.

Day of ovarian stimulation	Correlations between urine estradiol level with variables						
	Number of follicles	Number of follicles size	Number of oocytes retrieved	Number of metaphase II oocytes	2PN stage	Cleavage stage	Blastocyst stage
Day 6th of ovarian stimulation rho (95% CI)	0.72 (0.59–0.81)	0.39^a^ (0.18–0.54)	0.42 (0.22–0.59)	0.44 (0.24–0.60)	0.47 (0.28–0.63)	0.43 (0.23–0.60)	0.48 (0.29–0.64)
Day of ovarian trigger rho (95% CI)	0.78 (0.64–0.85)	0.57^b^ (0.40–0.70)	0.58 (0.41–0.71)	0.61 (0.45–0.73)	0.64 (0.49–0.76)	0.62 (0.46–0.74)	0.57 (0.40–0.70)

*PN* pronuclei.

^a^Correlation between urine E2 and Number of follicles size ≥ 10.

^b^Correlation between urine E2 and Number of follicles size ≥ 14.

The sub-analysis presented that on the day 6th of ovarian stimulation, women who had an age ≥ 35 years the urine E2 level 0.72 (*p* < 0.001) was significantly correlated when compared to women with age < 35 years 0.35 (*p* = 0.18). While on the day of ovarian trigger, the urine E2 levels and serum E2 levels were significantly correlated in both age groups: 0.52 in age < 35 years and 0.60 in age ≥ 35 years. Moreover, the urine E2 level was moderately correlated in a group of women with normal BMI (0.50, *p* < 0.001) and high BMI (0.54, *p* < 0.001) while low the BMI group was lowly correlated (0.13, *p* = 0.39) on day 6. Furthermore, the urine E2 levels in the normal responder group were significantly correlated with serum E2 levels on day 6 of ovarian stimulation (0.82, *p* < 0.001) compared to the poor and high responder groups. In contrast, on the day of ovarian trigger, the poor responding group had a statistically significant difference in the correlation between the urine and serum E2 levels (0.42, *p* < 0.05) when compared to the other groups (Table 4).

**Table 4:** Spearman correlations of urine and serum estradiol in subgroup analysis.

Variables	Day 6 of ovarian stimulation (95% CI)	*p* value	Day of ovarian trigger (95% CI)	*p* value
**Age**				
Age < 35 years (n = 32)	0.35 (0.00–0.62)	0.18	0.52 (0.21–0.74)	< 0.05
Age ≥ 35 years (n = 45)	0.72 (0.54–0.84)	< 0.001	0.60 (0.37–0.76)	< 0.05
**BMI**				
Low BMI (n = 7) (BMI < 18.5 kg/mm^3^)	0.29 (− 0.59 to 0.86)	0.53	0.79 (0.09–0.97)	< 0.05
Normal BMI (n = 45) (BMI 18.5–24.9 kg/mm^3^)	0.50 (0.24–0.69)	< 0.001	0.59 (0.40–0.75)	< 0.001
High BMI (n = 25) (BMI > 25 kg/mm^3^)	0.54 (0.19–0.77)	< 0.05	0.53 (0.71–0.76)	< 0.05
**Ovarian response**				
Poor response (n = 43) (number of retrieve oocyte < 10)	0.13 (− 0.18 to 0.41)	0.39	0.42 (0.14–0.64)	< 0.05
Normal response (n = 17) (number of retrieve oocyte 10–20)	0.82 (0.56–0.93)	< 0.05	0.35 (− 0.16 to 0.71)	0.28
High response (n = 23) (number of retrieve oocyte > 20)	0.32 (− 0.11 to 0.65)	0.14	0.11 (− 0.32 to 0.60)	0.61

The correlations between serum E2 at 0.72 and sonographic follicles on the 6th day and the last day of ovarian stimulation were 0.78 (*p* < 0.001), which showed a higher correlation than urine E2 and sonographic follicles.

There were not any COH complications such as OHSS or oocyte retrieval such as bleeding or infection. The specimens were collected and analyzed without any mistakes or complications.

## Discussion

In this study, it was found that the correlations of the relative levels of serum and urine E2 were valid on both on the 6th day of ovarian stimulation and the day of ovarian trigger in infertile women who had undergone the COH cycle. There was a moderate correlation between serum and urine E2 concentration on the 6th day of ovarian stimulation (r = 0.53) and on the day of ovarian trigger (r = 0.59). The positive linear correlation in urine E2 levels on the day of ovarian trigger and number of follicle sizes (size ≥ 10 mm r = 0.57), number of retrieved oocytes, r = 0.58, number of metaphase II oocytes, r = 0.61 and rate of fertilization, r = 0.64, were relatively significant. Moreover, subgroup analysis was done for evaluation between serum and urine E2 level in factors of IVF success rate (age, BMI, and responding status) and the results showed that there was a significant correlation in women age ≥ 35 year and normal to high BMI, while this correlation was uncertain in the responding factors.

This study results are consistent with previous studies. A 1981 reported by Miyakawa et al.^14^ reported that the urinary estradiol-17 beta-glucuronide (E2-17G) measured by direct radioimmunoassay (RIA) correlation was significantly correlated with serum estrogens in women treated with gonadotropin on the day of ovarian trigger. Frenken et al.^15^ reported that total the excretion per 24-h or the concentrations per liter in the 24-h urine collection of estrogen and estrone-3-glucorunide on the day of prior ovarian trigger were in good correlation with serum E2 levels in women treated with HMG. In addition, a 1992 study by Rapi et al. used a chemiluminescence immunoassay (LIA) method in early morning urine (EMU) samples to evaluate the correlation between serum E2 level and urine E1-3G level on the day of ovarian trigger. They revealed that the urine E1-3G excretions were consistent with serum E2 levels in women treated with COH^16^. In the clinical setting, RIA is a complex method that is performed using a 24-h collection. For simplicity, the ECLIA method was used to evaluate spot urine which is convenient for analyses urine samples. Furthermore, the ECLIA showed good sensitivity in normal and high E2 level women and is used worldwide^17,18^.

The present study is the first study that evaluates the correlation between urine E2 levels with the number of follicle growth, number of retrieved oocytes, number of metaphase II oocytes and rate of fertilization with which these data were associated with the IVF outcomes. From these results, we assumed that urine E2 level on the 6th day of stimulation might be a predictor of the mean number of oocytes growth. In addition, urine E2 levels on the day of ovarian trigger might also predict, the mean numbers of retrieval follicles, numbers of MII oocytes and fertilization rate.

We performed sub-analysis to investigate the factors that might affect the outcomes especially, age, BMI and responding status. We found that some factors were slightly impact on the outcomes such as age and BMI. In current literature reviews, there are not information to explain the effect of the age or BMI on the correlation between serum and urine E2 level. Therefore, we need larger sample size and randomized controlled studies to confirm these effects.

To the best of the authors knowledge, this is the first study that used ECLIA which a good sensitivity method to evaluate the urine E2 level. Using urine samples to monitor E2 level is more convenient and simplifies collection and analysis when compared to serum. In addition, the fact that the serum and urine E levels were collected on the same day reduced possible confounding factors from the fluctuations of hormone levels, moreover, urine E2 levels from collected urine E2 to creatinine ratio reduced the effect of the glomerular infiltration on urine E2 excretion. The limitation of this study, other hormones such follicle stimulating hormone (FSH), luteinizing hormone (LH) and progesterone that might affect to IVF outcomes were not evaluated. Furthermore, some hormones especially LH were unstable and had short half-life in the plasma.

Based on the results of this study, the urine E2 level was correlated with serum E2 level in COH stimulation on the 6th day of ovarian stimulation and the day of ovarian trigger. It may be an advantage to implement this collection method during COH especially in the cases where it is inconvenient to collect serum samples. Further study is needed to confirm the efficacy of this method in women with the risk factor of OHSS.

In conclusion, the urine E2 level was moderately correlated to serum E2, follicle growth, retrieved oocytes and also fertilization rate. We assumed that the urine E2 might be the alternative test for ovarian stimulation monitoring in women who cannot collect blood specimen such as obesity women and anxiety women.

## Data Availability

Data and materials are available upon request.
